# Supplementary material for: Local adaptation and archaic introgression shape global diversity at human structural variant loci
Source: eLife. 2021 Sep 16;10:e67615. doi: 10.7554/eLife.67615 (PMC8492059; doi:10.7554/eLife.67615)
Supplement: Supplementary file 1. — The top three SVs per ancestry component are reported. The IGH insertion and deletion are highlighted in bold text. [file elife-67615-supp1.docx]

| **SV ID** | **SV length** | **LRS** | ***P*-value** | **Ancestry component** | **Affected gene(s)** |
| --- | --- | --- | --- | --- | --- |
| 27407_HG02106_ins | 44 | 124.3 | 2.74 x 10^-23^ | 1 |  |
| 1737_HG02106_del | 3490 | 58.5 | 7.69 x 10^-9^ | 1 | *LINC01680* |
| 11915_HG01352_ins | 397 | 45.0 | 7.28 x 10^-6^ | 1 | *KDM2A* |
| **22237_HG02059_ins** | **34** | **513.0** | **5.34 x 10^-108^** | **2** | ***IGHG4*** |
| **22231_HG02059_del** | **135** | **488.1** | **1.42 x 10^-102^** | **2** |  |
| 25871_AK1_del | 37 | 113.7 | 5.77 x 10^-21^ | 2 |  |
| 32021_HG00268_ins | 84 | 98.9 | 1.02 x 10^-17^ | 3 | *PCNT* |
| 2181_CHM1_ins | 309 | 84.6 | 1.36 x 10^-14^ | 3 | *TMEM131* |
| 1731_NA12878_ins | 395 | 82.2 | 4.57 x 10^-14^ | 3 | *LRP1B* |
| 22065_HG02106_del | 186 | 481.1 | 4.67 x 10^-101^ | 4 |  |
| 25687_HG02106_ins | 2886 | 468.6 | 2.39 x 10^-98^ | 4 | *CLEC16A* |
| 5843_HG02106_del | 32 | 377.3 | 1.82 x 10^-78^ | 4 |  |
| 21859_NA19240_ins | 36 | 159.1 | 6.70 x 10^-31^ | 5 | *AC135050.3* |
| 25014_HG02106_del | 325 | 133.4 | 2.80 x 10^-25^ | 5 | *CSNK1G1, AC087632.2* |
| 21191_NA19240_del | 110 | 122.1 | 8.12 x 10^-23^ | 5 | *AC087632.2, PCLAF* |
| **22237_HG02059_ins** | **34** | **212.2** | **1.73 x 10^-42^** | **6** | ***IGHG4*** |
| 658_HX1_ins | 445 | 137.6 | 3.42 x 10^-26^ | 6 |  |
| **22231_HG02059_del** | **135** | **135.9** | **7.80 x 10^-26^** | **6** |  |
| 10085_HG00268_del | 52 | 116.6 | 1.31 x 10^-21^ | 7 |  |
| 18105_NA19240_ins | 3096 | 107.7 | 1.21 x 10^-19^ | 7 |  |
| 23087_CHM13_del | 34 | 102.3 | 1.78 x 10^-18^ | 7 | *PSMC3IP* |
| 18075_HG00268_del | 167 | 85.6 | 8.43 x 10^-15^ | 8 | *PLXDC2* |
| 9365_CHM1_ins | 4417 | 83.9 | 1.99 x 10^-14^ | 8 | *CLVS1* |
| 14974_NA19434_ins | 337 | 63.6 | 5.64 x 10^-10^ | 8 | *OR10G3* |

**Supplementary File 1.** Highly differentiated SV loci across ancestry components. The top three SVs per ancestry component are reported. The *IGH* insertion and deletion are highlighted in bold text.

­­­­
