# Supplementary material for: Local adaptation and archaic introgression shape global diversity at human structural variant loci
Source: eLife. 2021 Sep 16;10:e67615. doi: 10.7554/eLife.67615 (PMC8492059; doi:10.7554/eLife.67615)
Supplement: Supplementary file 2. [file elife-67615-supp2.docx]

­­­­­­

| **SV ID** | **LRS** | **Ancestry component** | **eQTL gene** | **eQTL beta perm. p-value** |
| --- | --- | --- | --- | --- |
| 10847_AK1_del | 81.2 | 2 | *SPDYE18* | 2.9 x 10^-3^ |
| 1400_HG00268_del | 49.6 | 2 | *IL6R* | 2.1 x 10^-3^ |
| 18458_HG01352_del | 61.2 | 2 | *ZNF295-AS1* | 2.6 x 10^-4^ |
| 20576_CHM13_ins | 59.3 | 2 | *RP11-578F21.9* | 3.3 x 10^-3^ |
| 21859_NA19240_ins | 159.1 | 4 | *MIR4519* | 2.8 x 10^-6^ |
| 22622_HG04217_del | 53.0 | 2 | *RP11-511H23.2* | 3.7 x 10^-3^ |
| 46317_HG04217_del | 86.7 | 6 | *RP11-848P1.7* | 3.4 x 10^-3^ |

**Supplementary File 2.** Highly differentiated SVs that are also significant eQTLs.
