# Supplementary material for: Local adaptation and archaic introgression shape global diversity at human structural variant loci
Source: eLife. 2021 Sep 16;10:e67615. doi: 10.7554/eLife.67615 (PMC8492059; doi:10.7554/eLife.67615)
Supplement: Supplementary file 3. — The IGH insertion and deletion are highlighted in bold text. [file elife-67615-supp3.docx]

| **SV ID** | **Max r^2^ with Sprime SNP** | **LRS** | **Ancestry component** | **Affected gene(s)** |
| --- | --- | --- | --- | --- |
| 16140_HX1_ins | 1.00 | 86.9 | 6 |  |
| 16624_HG00268_del | 1.00 | 53.8 | 3 |  |
| 1929_HG02106_del | 1.00 | 173.4 | 4 | *KDM2A* |
| 20771_CHM13_del | 1.00 | 223.1 | 4 | *TCF12* |
| **22231_HG02059_del** | **1.00** | **135.9** | **6** |  |
| 24577_HG00514_del | 1.00 | 109.9 | 2 | *TCF25* |
| 29991_HG02106_del | 1.00 | 200.5 | 4 |  |
| 3098_HG00268_ins | 1.00 | 50.0 | 3 |  |
| 41325_HG04217_del | 1.00 | 100.3 | 5 |  |
| 8362_HG02106_ins | 1.00 | 189.6 | 4 |  |
| 19726_CHM13_ins | 0.98 | 177.8 | 4 |  |
| 10377_HG02106_ins | 0.97 | 101.1 | 6 | *FBXO38* |
| 16625_HG00268_ins | 0.97 | 53.4 | 3 |  |
| 20769_CHM13_ins | 0.96 | 225.8 | 4 | *TCF12* |
| 18686_HG01352_ins | 0.96 | 77.1 | 6 |  |
| 10847_AK1_del | 0.96 | 81.2 | 3 | *AC007000.4* |
| 12550_HG00733_ins | 0.94 | 36.8 | 1 | *LINC00301* |
| 10373_HG02106_ins | 0.92 | 171.6 | 4 |  |
| **22231_HG02059_del** | **0.88** | **488.1** | **2** |  |
| 7453_HG02059_ins | 0.83 | 74.1 | 2 |  |
| **22237_HG02059_ins** | **0.78** | **513.0** | **2** | ***IGHG4*** |
| 22237_HG02059_ins | 0.78 | 513.0 | 6 | *IGHG4* |
| 12546_HG00733_ins | 0.78 | 32.7 | 1 | *MS4A19P* |
| 32432_HG00268)del | 0.67 | 153.9 | 4 |  |
| **22237_HG02059_ins** | **0.64** | **212.2** | **6** | ***IGHG4*** |
| 24574_HG00514_ins | 0.62 | 95.0 | 2 | *SPIRE2* |
| 6305_CHM13_del | 0.58 | 91.5 | 6 | *TLR1* |
| 13529_AK1_ins | 0.55 | 71.6 | 6 |  |
| 5444_HG00514_ins | 0.51 | 88.4 | 2 | *NEPRO* |

**Supplementary File 3.** Highly differentiated SVs in LD (*r*^2^ > 0.5) with putative archaic introgressed haplotypes called by Sprime. The *IGH* insertion and deletion are highlighted in bold text.
