# Supplementary material for: Local adaptation and archaic introgression shape global diversity at human structural variant loci
Source: eLife. 2021 Sep 16;10:e67615. doi: 10.7554/eLife.67615 (PMC8492059; doi:10.7554/eLife.67615)
Supplement: Transparent reporting form [file elife-67615-transrepform1.docx]

***eLife’s* transparent reporting form**

We encourage authors to provide detailed information *within their submission* to facilitate the interpretation and replication of experiments. Authors can upload supporting documentation to indicate the use of appropriate reporting guidelines for health-related research (see [EQUATOR Network](http://www.equator-network.org/%20)), life science research (see the [BioSharing Information Resource](https://biosharing.org/)), or the [ARRIVE guidelines](http://www.plosbiology.org/article/info:doi/10.1371/journal.pbio.1000412) for reporting work involving animal research. Where applicable, authors should refer to any relevant reporting standards documents in this form.

If you have any questions, please consult our Journal Policies and/or contact us: [editorial@elifesciences.org](mailto:editorial@elifesciences.org).

**Sample-size estimation**

- You should state whether an appropriate sample size was computed when the study was being designed
- You should state the statistical method of sample size computation and any required assumptions
- If no explicit power analysis was used, you should describe how you decided what sample (replicate) size (number) to use

Please outline where this information can be found within the submission (e.g., sections or figure legends), or explain why this information doesn’t apply to your submission:

The entire set of 2504 unrelated samples of the 1KGP dataset were included in our graph-genotyping analysis. For eQTL mapping, we used the entire set of 447 intersecting samples from the Geuvadis dataset that are also part of the unrelated 1KGP dataset. While no explicit power analysis was conducted, we used the entirety of these available datasets to maximize statistical power. These samples are described at the beginning of the respective sections of the Methods.

**Replicates**

- You should report how often each experiment was performed
- You should include a definition of biological versus technical replication
- The data obtained should be provided and sufficient information should be provided to indicate the number of independent biological and/or technical replicates
- If you encountered any outliers, you should describe how these were handled
- Criteria for exclusion/inclusion of data should be clearly stated
- High-throughput sequence data should be uploaded before submission, with a private link for reviewers provided (these are available from both GEO and ArrayExpress)

Please outline where this information can be found within the submission (e.g., sections or figure legends), or explain why this information doesn’t apply to your submission:

No new raw data were generated by our study. Details of inclusion/exclusion criteria for quality-control of structural variant genotypes are described and justified in the corresponding section of the Methods. All 1KGP and Geuvadis samples are biological replicates. Links to all datasets used in our study can be found upon first mention in the Methods, while a link to our analysis code, data summaries, and results can be found in the Data and Software Availability section.

**Statistical reporting**

- Statistical analysis methods should be described and justified
- Raw data should be presented in figures whenever informative to do so (typically when N per group is less than 10)
- For each experiment, you should identify the statistical tests used, exact values of N, definitions of center, methods of multiple test correction, and dispersion and precision measures (e.g., mean, median, SD, SEM, confidence intervals; and, for the major substantive results, a measure of effect size (e.g., Pearson's r, Cohen's d)
- Report exact p-values wherever possible alongside the summary statistics and 95% confidence intervals. These should be reported for all key questions and not only when the p-value is less than 0.05.

Please outline where this information can be found within the submission (e.g., sections or figure legends), or explain why this information doesn’t apply to your submission:

Statistical analysis decisions are described and justified in either the Results or Methods section, as appropriate. For example, we describe the Ohana approach (based on a likelihood ratio test) at a high level in the Results and in greater detail in the Methods, while also referencing the original paper with the full methodological description. Statistical details (including normalization, definition of the cis-window, multiple testing correction, etc.) of eQTL mapping are similarly provided in the Methods section. No figures include fewer than 10 data points, but visualizations have been chosen with a goal of maximizing interpretability and transparency. Raw output of both the selection scan (with Ohana) and eQTL mapping (with FastQTL) are uploaded to the Zenodo repository described in the Data and software availability section. These include the test statistics, standard errors, and p-values for all tested variants. Exact p-values are reported throughout the manuscript. Point estimates and credible intervals are reported for all parameter estimates in the section describing modelling of the selection event in the results section.

(For large datasets, or papers with a very large number of statistical tests, you may upload a single table file with tests, Ns, etc., with reference to sections in the manuscript.)

**Group allocation**

- Indicate how samples were allocated into experimental groups (in the case of clinical studies, please specify allocation to treatment method); if randomization was used, please also state if restricted randomization was applied
- Indicate if masking was used during group allocation, data collection and/or data analysis

Please outline where this information can be found within the submission (e.g., sections or figure legends), or explain why this information doesn’t apply to your submission:

For the selection scan with Ohana, we did not pre-define populations, but instead performed global ancestry component inference with Ohana. This approach and rationale (including the choice of k) is described in detail in the Results and Methods sections. For the demographic modelling section, we used the population assignments provided by 1KGP, as described in detail in the Methods section.

**Additional data files (“source data”)**

- We encourage you to upload relevant additional data files, such as numerical data that are represented as a graph in a figure, or as a summary table
- Where provided, these should be in the most useful format, and they can be uploaded as “Source data” files linked to a main figure or table
- Include model definition files including the full list of parameters used
- Include code used for data analysis (e.g., R, MatLab)
- Avoid stating that data files are “available upon request”

Please indicate the figures or tables for which source data files have been provided:

Source data files have now been provided for Fig. 1C, Fig. 2, Fig. 3A, and Fig. 5D-G. All analysis code for reproducing all figures, as well as larger data summaries and results can be found in the Data and Software Availability section.
